# Endometriosis‐derived exosomes encapsulated miR‐196a‐5p mediate macrophage polarization through regulation of the Hippo pathway

**DOI:** 10.1002/ccs3.70020

**Published:** 2025-05-25

**Authors:** Bin Lu, Qixiang Huang, Yanyu Zhong

**Affiliations:** ^1^ Department of Gynecology Wuhu No.1 People's Hospital Wuhu City China; ^2^ Department of Obstetrics and Gynecology The First Affiliated Hospital of Nanchang University Nanchang China; ^3^ Reproductive Medicine Centre The First Affiliated Hospital of Soochow University Suzhou Jiangsu Province China

**Keywords:** endometriosis, exosome, Hippo pathway, macrophage, miR‐196a‐5p

## Abstract

Endometriosis (EMs) is a disease that adversely affects women's health. Immune imbalance is an important factor contributing to EMs, and exosomes (Exo) play an important role in immunomodulation. The purpose of this study was to investigate the effect of exosomes derived from the blood of patients with EMs on macrophage polarization and elucidate the underlying mechanisms. Exosomes were isolated from the serum of healthy controls (control exosomes) and patients with EMs (EMs exosomes). Macrophage polarization levels were detected using flow cytometry (FCM), RT‐qPCR, and Western blot. Subsequently, we used RNA sequencing to analyze differential microRNAs (miRNA) and associated pathways. Electroporation techniques were used to modify the exosomes. The associated pathways were analyzed by Western blot. Finally, 12Z cells were co‐cultured with macrophages of different polarizations, and the viability and metastasis of 12Z cells were calculated by cell counting kit‐8 (CCK‐8), scratch, and Transwell. EMs exosomes induced M2‐type polarization in macrophages. RNA sequencing results showed that miR‐196a‐5p was dramatically decreased in EMs exosomes, whereas overexpression of miR‐196a‐5p in EMs exosomes could inhibit the M2‐type polarization of macrophages and activate the Hippo pathway. In addition, M2‐type macrophages promoted 12Z cell proliferation and metastasis. These findings suggest that serum‐derived exosomes encapsulating miR‐196a‐5p alleviate endometriosis by promoting M1‐type macrophage polarization via Hippo pathway activation.

## Graphical abstract

Encapsulation of miR‐196a‐5p mimic in serum‐derived exosomes from patients with endometriosis by electrotransfection: exosomes encapsulated with miR‐196a‐5p are phagocytosed by macrophages and release miR‐196a‐5p; miR‐196a‐5p activates the Hippo pathway and promotes macrophage M1‐type polarization, thereby inhibiting 12Z cell metastasis.

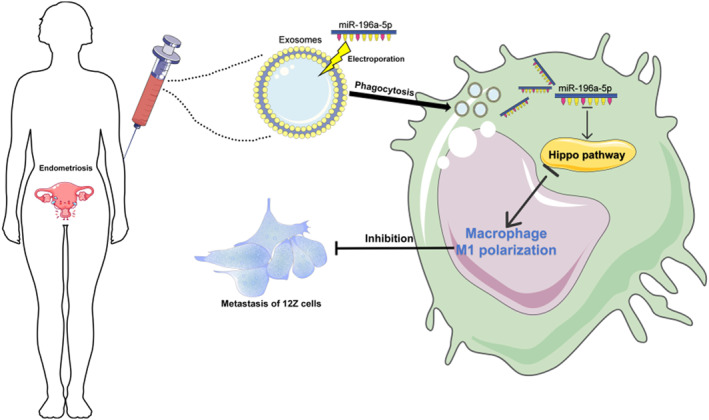

## 1 INTRODUCTION

Endometriosis (EMs) is characterized by the growth of functional endometrial tissue outside the uterine cavity.^1^ It is a common, treatment‐resistant, benign gynecological disorder frequently occurring in women of childbearing age. Although EMs is a benign disease, it exhibits aggressive behavior, including invasion, planting, metastasis, recurrence, and malignancy.^2^ Its main symptoms include secondary dysmenorrhea with progressive worsening, chronic pelvic pain, and infertility; these symptoms significantly impair patients' quality of life.^3^

The pathogenesis of endometriosis remains unclear, with multiple hypotheses proposed. In past years, several findings have suggested that an imbalance in the local immune microenvironment is a prominent factor in the process of endometriosis.^4^, ^5^, ^6^ In particular, macrophages are activated as inflammatory cells in the disease process of endometriosis, suggesting that macrophages are likely to be involved in and regulate the development of the endometriosis disease process.^7^

Exosomes, which are microvesicles with a diameter ranging from 50 to 150 nm and are secreted by various cells, mainly exhibit a double‐membrane structure resembling a teatox.^8^ Exosomes play a prominent role in the extracellular environment as interconnections between cells. Initially, exosomes were considered cellular waste products.^9^ In recent years, however, studies have shown that exosomes are capable of carrying important substances, such as nucleic acids, lipids, and proteins, making exosomes an important factor in cellular interactions.^10^ Sahar and his colleagues reported that exosomes are involved in the course of a variety of female reproductive disorders and could serve as key markers in the diagnosis and treatment of reproductive diseases.^11^ In addition, Zhang et al.^12^ reported that exosomes regulated endometrial cell apoptosis and inflammation through the VEGF/NF‐κB signaling pathway. Interestingly, Huang et al.^13^ similarly found that miR‐301a‐3p in endometriosis patient‐derived exosomes promotes macrophage polarization involved in the endometriosis process. These studies invariably demonstrated that exosomes play a crucial role in endometriosis.

MicroRNAs (miRNAs), a class of noncoding RNAs, are widely expressed in a variety of cells and tissues.^14^ miRNAs are involved in the regulation of numerous biological processes, including inflammation, tissue repair, and immune homeostasis.^15^ miR‐196a‐5p has been reported to play a significant role in several diseases. For instance, He et al.^16^ reported that miR‐196a‐5p enhanced epithelial‐mesenchymal transition in colorectal cancer cells, facilitating cancer metastasis. Fei et al.^17^ identified miR‐196a‐5p in exosomes secreted by tumor fibroblasts as a new potential target for lung cancer because of its ability to enhance the radiation resistance of lung cancer cells. Furthermore, miR‐196a‐5p in exosomes secreted by trophoblast cells promotes macrophage M1‐type polarization during the pathogenesis of recurrent abortion.^18^ The above studies amply demonstrated that miR‐196a‐5p is involved in a variety of diseases, including female obstetric diseases, and has an effect on macrophage polarization. However, the interaction between miR‐196a‐5p and macrophages in the development of endometriosis remains unknown.

In summary, further in‐depth investigation of the interactions among exosomes, miRNAs, and macrophages is essential for the development of novel diagnostic and therapeutic strategies for endometriosis. Therefore, this study aimed to determine the effect of exosomes containing miR‐196a‐5p on macrophage polarization and elucidate the underlying molecular mechanisms in serum‐derived exosomes from patients with endometriosis.

## 2 MATERIALS AND METHOD

### 2.1 Clinical specimens

This study was conducted in accordance with the ethical guidelines approved by the Ethics Committee of Wuhu No. 1 People's Hospital (No. YYLL20220089), and written informed consent was obtained from all participants. Blood from healthy women and patients with confirmed endometriosis was collected in vacuum blood collection tubes and left to stand for 30 min, followed by centrifugation at 2500 rpm for 15 min to separate the serum. This study included 10 samples: 5 healthy controls and 5 patients with endometriosis. All patients (28 ± 6 years) underwent laparoscopic examination of the pelvis and adjacent areas, which was corroborated by histopathologic testing to affirm the diagnosis of endometriosis. The histopathologic diagnostic criteria included the presence of endometrial glands and mesenchymal stroma within foci characterized by inflammatory responses and fibrosis. Healthy control group donors (29 ± 7 years) were women whose physical examination showed that they were healthy and free of endometriosis. Inclusion criteria for this study were as follows: (i) diagnosis of endometriosis confirmed by laparoscopy and histopathological testing; (ii) no hormonal therapy for 6 months prior to collection of samples; and (iii) consent and acceptance for inclusion in the study. Exclusion criteria were as follows: (i) patients with hemocytolysis at the time of the examination and (ii) patients who did not agree to be included in the study.

### 2.2 Exosome isolation and identification

Isolation of exosomes was performed using a rapid exosome extraction kit (41202ES30, YEASEN, China) for serum samples according to the manufacturer's instructions. Briefly, serum samples were added to the premix provided in the kit and centrifuged to obtain a precipitate, which was resuspended in PBS to clean the precipitate and centrifuged again to collect the supernatant, which was rich in exosomes. The morphology of exosomes was identified using transmission electron microscopy (Spectra 300 S, Thermo Fisher Scientific, USA), and the particle size was analyzed and identified by the NTA system (NS300, Malvern Panalytical, UK). Exosome marker proteins were identified by Western blot.

### 2.3 Cell culture and induction

THP‐1 cells, human acute monocytic leukemia cells, were purchased from Sunncell Biologicals Ltd. (SNL‐044, Wuhan, China). The THP‐1 cell line was authenticated by short tandem repeat (STR) profiling and tested negative for mycoplasma contamination. The cells were cultivated in RPMI 1640 medium (41402ES76, YEASEN, China) containing 10% FBS (fetal bovine serum, 40131ES76, YEASEN, China), 1% streptomycin‐penicillin (P/S, 60162ES76, YEASEN, China), and 0.05 mM β‐mercaptoethanol (C7122, Bioss, China) at 37°C with 5% CO_2_. 12Z cells (immortalized human endometriosis cells) were cultured in DMEM/F12 medium (41420ES76, YEASEN, China) containing 10% FBS and 1% P/S, and the cells were provided by ZQXZBIO. THP‐1 cells were differentiated into macrophages using 100 ng/mL PMA.

When THP‐1 cells were induced into macrophages by PMA, macrophages were treated with exosomes (20 μg/mL) for 24 h, and cell samples were collected for subsequent experiments. XMU‐MP‐1 (1 mM, S8334, Selleck, China) was used as a Hippo pathway inhibitor for treatment of macrophages.

### 2.4 PKH26 labeling

Exosomes were labeled with the PKH26 fluorescent probe (40780ES20, YEASEN, China) and co‐cultured (20 μg/mL) with THP‐1‐derived macrophages. After 24 h, the macrophage was labeled with DAPI (C1005, Beyotime, China). The uptake of exosomes by macrophages was observed using fluorescence microscopy (NIB1000, Novel, China).

### 2.5 Small RNA sequencing of exosomes

After the exosomes were extracted and identified correctly, total RNA was extracted from the exosomes, and high‐throughput sequencing was performed using the Illumina NovaSeq 6000 platform. Briefly, after RNA extraction and identification, library construction was performed using the Sample Pre Kit. After the library construction was completed, the effective concentration of the library was accurately quantified using RT‐qPCR to ensure the quality of the library. After the library was qualified, sequencing was performed using the Illumina NovaSeq 6000 platform.

### 2.6 Detection of macrophages polarization

The proportion of differently polarized macrophages was determined by flow cytometry. After various treatments, macrophages were collected and fixed with Fixation Buffer (40402ES50, YEASEN, China) for 30 min. The fixative was then discarded, and the cells were washed twice with PBS for 5 min each. Subsequently, the cells were resuspended in flow cytometry buffer containing fluorescence‐conjugated antibodies against CD86 (an M1 marker, 374210, BioLegend, USA) and CD206 (an M2 marker, 321120, BioLegend, USA) and incubated at room temperature in the dark for 30 min. Finally, the stained cells were analyzed using a flow cytometer (NovoCyte Advanteon, Agilent, USA).

### 2.7 Electroporation

In order to modify exosomes, the miR‐196a‐5p mimic (5′‐UAGGUAGUUUCAUGUUGUUGGG‐3′), synthesized by RiboBio, was introduced into exosomes using the Neon NxT Electroporation System (Thermo Fisher Scientific, USA). The electroporation conditions were set to 1000 V, 10 ms, and 2 pulses. A total of 20 μg of exosomes was premixed with 200 nM of miR‐196a‐5p mimic in a buffer provided by the Neon kit (N1025, Thermo Fisher Scientific, USA), with a volume of 200 μL. The mixture was thoroughly homogenized and transferred to an electroporation tube, which was then placed in the electroporator for electroporation.

### 2.8 Quantitative real‐time PCR

Total RNA was extracted from cells using TRIzol reagent (10606ES60, YEASEN, China). cDNA was synthesized from the extracted RNA using a reverse transcription kit (R223, Vazyme, China). The amplification system was configured according to the instructions of the SYBR green amplification kit (KCQS00, Sigma, USA), and the amplification detection was performed on the RT‐qPCR instrument system (CFX96^TM^, Bio‐Rad, USA). U6 and β‐actin were used as internal reference genes. The primers are referenced in Table 1.

**TABLE 1:** Primer for RT‐qPCR.

Gene	Primer (5′‐3′)
miR‐196a‐5p	F: CCGGCTAGGTAGTTTCATGTT
	R: CGGCCCAGTGTTCAGACTAC
U6	F: GCTTCGGCAGCACATATACTAAAAT
	R: CGCTTCACGAATTTGCGTGTCAT
iNOS	F: TTCAGTATCACAACCTCAGCAAG
	R: TGGACCTGCAAGTTAAAATCCC
CD86	F: CTCTGGTGCTGCTCCTCTGA
	R: TTCCTGGTCCTGCCAAAATA
CD206	F: GAAAGGTGACCCTACTATG
	R: AGTAACTGGTGGATTGTCTTG
CD163	F: GCGGGAGAGTGGAAGTGAAAG
	R: GTTACAAATCACAGAGACCGCT
β‐actin	F: CATGTACGTTGCTATCCAGGC
	R: CTCCTTAATGTCACGCACGAT

### 2.9 Protein extraction and Western blot

Total protein was extracted using RIPA protein lysis buffer (PC101, Epizyme, China), and the protein concentration was determined by the BCA quantification kit (ZJ101, Epizyme, China). 5 × loading buffer (LT103, Epizyme, China) was added to the protein samples, which were then heated at 100°C for 10 min to denature the proteins. The SDS‐PAGE electrophoresis gel was prepared according to the instructions of the Western Blot Kit (E‐IR‐R304A, Elabscience, China), and 30 μg of protein sample was added to each well for electrophoresis. After electrophoresis, the proteins were transferred onto a PVDF membrane (IPVH00010, Merck, USA). The PVDF membrane was then blocked with 5% skim milk (PS112, Epizyme, China), incubated overnight with the primary antibody, washed with PBS, and then incubated with the secondary antibody. Finally, protein bands were visualized using an ECL chemiluminescent reagent (SQ201, Epizyme, China) in an imaging system (SCG‐W2000, ServiceBio, China). Antibody information is listed in Table 2.

**TABLE 2:** Antibody for Western blot.

Name	Catalog	Brand	Dilution ratio
Calnexin	AF5362	Affinity	1/1000
TSG101	DF8427	Affinity	1/1000
CD63	AF5117	Affinity	1/500
iNOS	AF0199	Affinity	1/500
CD86	DF6332	Affinity	1/500
CD206	DF4149	Affinity	1/500
CD163	DF8235	Affinity	1/500
MST1	3682	CST	1/1000
p‐YAP1	13008	CST	1/1000
YAP1	14074	CST	1/1000
p‐TAZ	AF4315	Affinity	1/1000
TAZ	72804	CST	1/1000
β‐actin	4970	CST	1/5000
Goat anti‐rabbit IgG HRP	S0001	Affinity	1/3000

### 2.10 Co‐culture system

THP‐1 cells, after being induced into macrophages with PMA, were treated with different exosomes to induce different types of polarization. The macrophages were then seeded into the upper chamber of a co‐culture system (14151, Labselect, China), whereas 12Z cells were seeded into the lower chamber. The co‐culture was maintained for 24 h. Subsequently, the 12Z cells were collected for further experiments.

### 2.11 CCK‐8 assay

Cells were inoculated into a 96‐well plate, with each well containing approximately 5 × 10^3^ cells. After the cells adhered, the medium was replaced with 100 μL containing 10 μL of CCK‐8 solution (40203ES60, YEASEN, China), followed by a 30‐min incubation in the dark. The absorbance was measured using a spectrophotometer (Epoch2, Biotek, China).

### 2.12 Cell scratch assay

12Z cells were first seeded into the lower chamber of the co‐culture system and allowed to adhere until they formed a confluent monolayer. Using a 200 μL pipette tip, a straight scratch was created across the cell monolayer, and images of the initial scratch were captured immediately under a microscope (CKX53, Olympus, Japan). Subsequently, macrophages of different polarization types were seeded into the upper chamber and co‐cultured for 24 h. The cells were observed under a microscope and photographed to assess the degree of wound healing.

### 2.13 Invasion assay

Collect the 12Z cells after co‐culture and seed them into the upper chamber of a Transwell insert precoated with matrix gel (HY‐K6001, MCE, USA). Add medium containing 15% FBS to the lower chamber. Incubate in a cell culture incubator for 24 h. After incubation, fix with 4% paraformaldehyde (60536ES76, YEASEN, China), wash twice with PBS, and stain with crystal violet (60506ES60, YEASEN, China). After staining, carefully wipe away the cells that have not invaded from the upper membrane using a cotton swab. Observe and count the number of cells that have invaded the lower membrane under a microscope.

### 2.14 Statistical analysis

All data were presented as the mean ± standard deviation (SD) of triplicate experiments. Statistical analyses were performed using Prism GraphPad 10.1.2 software. One‐way analysis of variance (ANOVA) was employed to assess differences among multiple groups, whereas unpaired or paired two‐tailed Student's *t*‐tests were used for comparisons between two groups. *p* < 0.05 indicates statistical significance.

## 3 RESULTS

### 3.1 Identification of exosomes from clinical samples

**FIGURE 1:**
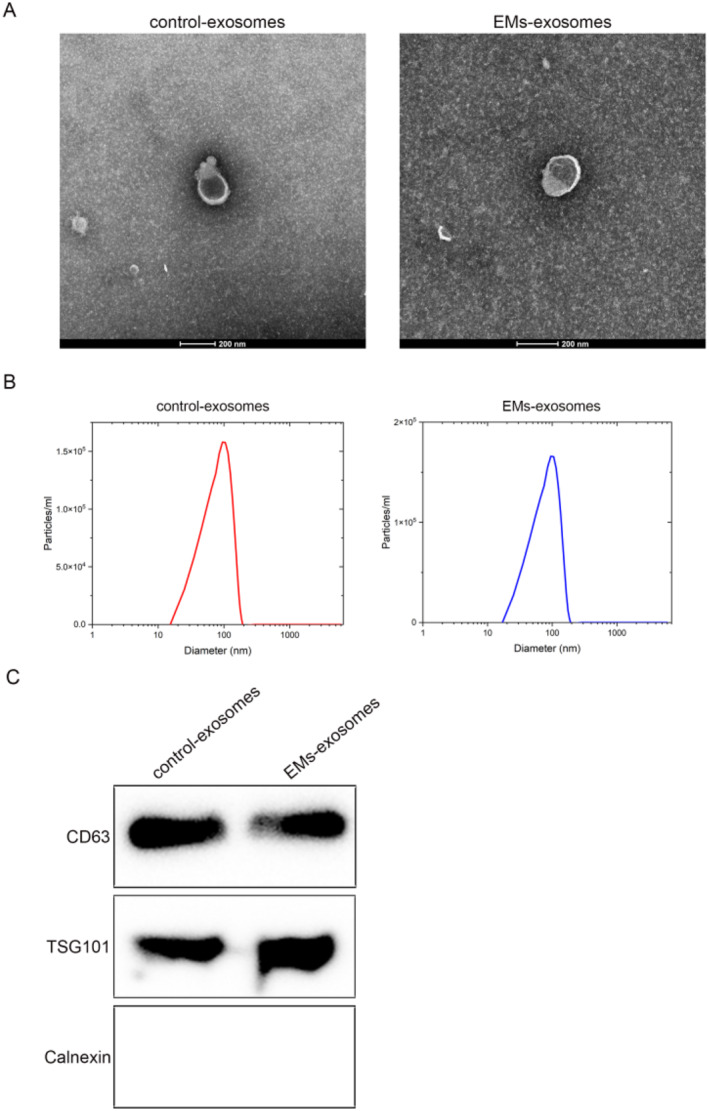
Characterization of exosomes. (A) The morphology of exosomes was detected using transmission electron microscopy (TEM) and TEM images showed the cup‐shaped, double‐membrane morphology of isolated exosomes (scale bar: 200 nm). (B) The particle size of exosomes was analyzed by the nanoparticle tracking analysis (NTA) system, and NTA demonstrated the size distribution of exosomes (peak diameter ∼100 nm). (C) Western blot was used to detect exosomal marker proteins (TSG101 and CD63) and the negative marker protein (calnexin), showing the expression of positive markers and the absence of the negative marker in exosomes. Data represent mean ± SD from three independent experiments. Control exosomes: serum‐derived exosomes from healthy individuals; EMs exosomes: serum‐derived exosomes from patients with endometriosis.

We collected serum from healthy controls and patients with endometriosis and isolated the exosomes from it. All exosomes were identified by TEM, NTA, and Western blot. TEM revealed that both control exosomes and EMs exosomes exhibited a cup‐shaped, double‐membrane structure (Figure 1A). NTA analysis results showed that the diameter of both groups of exosomes was approximately 100 nm (Figure 1B). The exosome‐positive marker proteins TSG101 and CD63 were expressed in both groups of exosomes, whereas the negative marker protein calnexin was not expressed in exosomes (Figure 1C). These results confirm successful exosome isolation.

### 3.2 EMs exosomes induce M2‐type polarization in macrophages

**FIGURE 2:**
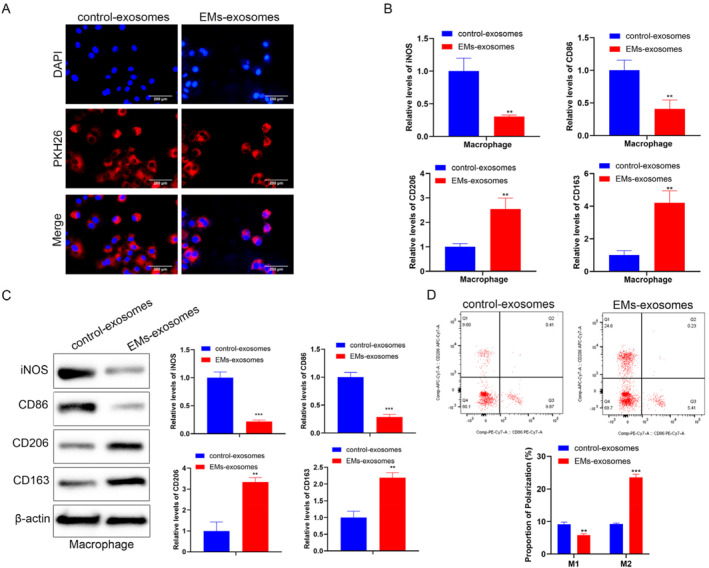
Role of EMs exosomes on macrophage polarization. (A) The exosomes were labeled with a PKH26 fluorescent probe and then co‐cultured with macrophages induced from THP‐1 cells, and the uptake of exosomes by macrophages was observed using a fluorescence microscope (scale bar: 200 μm). (B) RT‐qPCR was performed to detect the mRNA expression levels of macrophage polarization markers (iNOS and CD86 for M1 markers; CD206 and CD163 for M2 markers). (C) Western blot was used to detect the protein expression levels of macrophage polarization markers (iNOS and CD86 for M1 markers; CD206 and CD163 for M2 markers). (D) The proportion of CD86+ (M1) and CD206+ (M2) macrophages was detected using a flow cytometer. Data represent mean ± SD from three independent experiments. **: *P* < 0.01, ***: *P* < 0.001, compared with control exosomes.

To investigate the impact of EMs exosomes on macrophages, we treated the macrophages with exosomes. PKH26 staining demonstrated that macrophages internalized exosomes from both groups (Figure 2A). Compared with the control exosomes group, the expression of M1 macrophage polarization markers (iNOS and CD86) in the EMs exosomes group decreased, whereas the expression of M2 macrophage markers (CD206 and CD163) increased (Figure 2B,C). FCM results indicated that the proportion of M2 macrophages in the EMs exosomes group increased, and correspondingly, the proportion of M1 macrophages decreased (Figure 2D). These results suggest that exosomes derived from patients with endometriosis induce M2 polarization of macrophages.

### 3.3 Exosomal miRNA sequencing and bioinformatics

**FIGURE 3:**
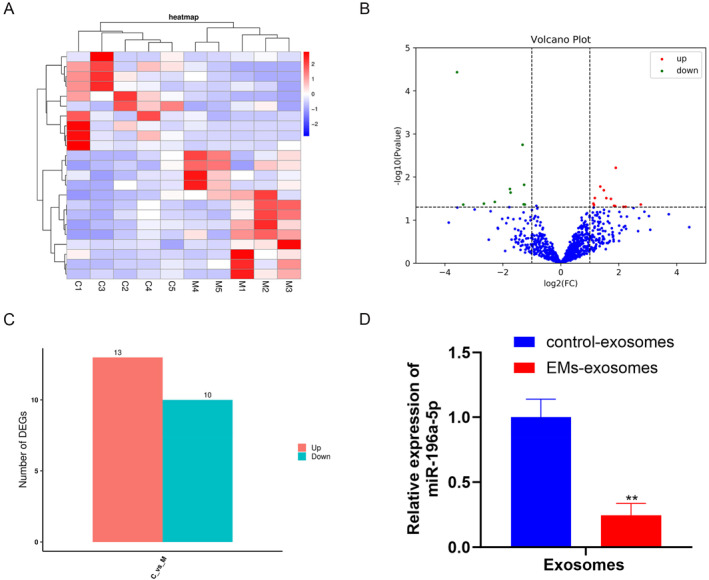
miRNA sequencing of exosomes. (A) Heatmap of differentially expressed miRNAs (red: upregulated; blue: downregulated). (B) Volcano map of differential miRNAs. (C) Statistical map of the number of differential miRNAs. (D) RT‐qPCR confirmation of miR‐196a‐5p downregulation in EMs exosomes. Data represent mean ± SD from three independent experiments. **: *P* < 0.01, compared with control exosomes.

To study the specific factors that induce macrophage polarization by exosomes, we extracted RNA from the two groups of exosomes for miRNA sequencing and subsequent analysis. The results in Figure 3A,B show that there were significant differences in miRNAs between the two groups of exosomes. Among them, sequencing analysis identified 13 miRNAs (hsa‐miR‐374a‐3p, hsa‐miR‐3135b, hsa‐miR‐6511a‐3p, hsa‐miR‐6131, hsa‐miR‐32‐5p, hsa‐miR‐29a‐5p, hsa‐miR‐4510, hsa‐miR‐3180‐3p, hsa‐miR‐6724‐5p, hsa‐miR‐590‐3p, hsa‐miR‐548e‐3p, hsa‐miR‐197‐5p, hsa‐miR‐6877‐5p) that were significantly increased and 10 miRNAs (hsa‐miR‐196a‐5p, hsa‐miR‐95‐3p, hsa‐miR‐335‐3p, hsa‐miR‐487a‐3p, hsa‐miR‐549a, hsa‐miR‐205‐5p, hsa‐miR‐548b‐5p, hsa‐miR‐675‐3p, hsa‐miR‐3117‐3p, hsa‐miR‐4417) that were significantly decreased in one group compared to the other (Figure 3C, Table 3). We identified a total of 23 miRNAs with differential expression; however, upon comparing the significance levels, we discovered that miR‐196a‐5p exhibited the lowest *p*‐value and the most pronounced difference. Notably, research on miR‐196a‐5p in the context of endometriosis is currently lacking. Consequently, our focus shifted toward miR‐196a‐5p. It was found through sequencing analysis that miR‐196a‐5p expression was significantly decreased in exosomes derived from endometriosis, and RT‐qPCR verification revealed that miR‐196a‐5p expression in EMs exosomes was significantly lower than that in control exosomes (Figure 3D). The above results suggest that the decreased expression of miR‐196a‐5p in EMs exosomes may be a factor influencing macrophage polarization.

**TABLE 3:** The list of all 13 miRNAs.

#ID	C	M	log2FC	Pvalue	Regulated
hsa‐miR‐196a‐5p	354.757	29.483	−3.582	0.000	Down
hsa‐miR‐95‐3p	628.389	251.483	−1.319	0.001	Down
hsa‐miR‐374a‐3p	38.507	144.005	1.895	0.006	Up
hsa‐miR‐335‐3p	599.227	248.341	−1.268	0.015	Down
hsa‐miR‐3135b	134.669	346.549	1.360	0.017	Up
hsa‐miR‐487a‐3p	36.820	10.318	−1.756	0.019	Down
hsa‐miR‐6511a‐3p	7.036	22.001	1.481	0.020	Up
hsa‐miR‐549a	13.316	3.736	−1.734	0.023	Down
hsa‐miR‐6131	120.124	272.572	1.172	0.031	Up
hsa‐miR‐32‐5p	4.905	13.778	1.569	0.031	Up
hsa‐miR‐29a‐5p	2.018	7.800	1.727	0.032	Up
hsa‐miR‐205‐5p	77.382	16.105	−2.278	0.038	Down
hsa‐miR‐4510	58.856	127.678	1.123	0.041	Up
hsa‐miR‐548b‐5p	3.174	0.402	−2.653	0.041	Down
hsa‐miR‐675‐3p	20.273	8.061	−1.284	0.043	Down
hsa‐miR‐3180‐3p	1.019	4.361	2.758	0.043	Up
hsa‐miR‐3117‐3p	14.391	6.430	−1.242	0.043	Down
hsa‐miR‐4417	4.208	0.258	−3.367	0.044	Down
hsa‐miR‐6724‐5p	5.873	13.872	1.142	0.044	Up
hsa‐miR‐590‐3p	2.751	10.737	1.835	0.046	Up
hsa‐miR‐548e‐3p	2.012	8.209	1.875	0.047	Up
hsa‐miR‐197‐5p	0.605	3.637	2.232	0.049	Up
hsa‐miR‐6877‐5p	1.589	8.020	2.158	0.049	Up

### 3.4 EMs exosomes encapsulate miR‐196a‐5p to inhibit M2 macrophage polarization

**FIGURE 4:**
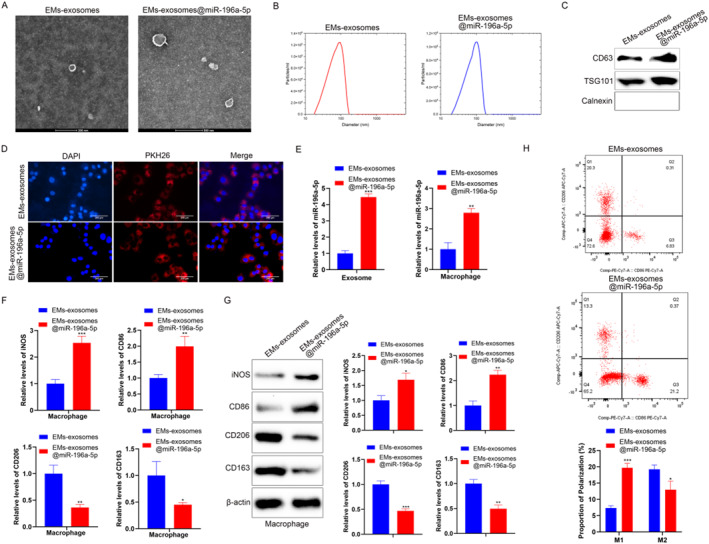
Effects of miR‐196a‐5p encapsulated in exosomes on macrophages. (A) EMs exosomes were electroporated with a miR‐196a‐5p mimic (5′‐UAGGUAGUUUCAUGUUGUUGGG‐3′) using the Neon NxT Electroporation System, and the morphology of both electroporated exosomes (EMs exosomes@miR‐196a‐5p) and nonelectroporated EMs exosomes were characterized by TEM. (B) The particle size of EMs exosomes and EMs exosomes@miR‐196a‐5p was analyzed by NTA. (C) Western blot was used to detect exosomal marker proteins (TSG101 and CD63) and the negative marker protein (calnexin) in EMs exosomes and EMs exosomes@miR‐196a‐5p. (D) The uptake of EMs exosomes and EMs exosomes@miR‐196a‐5p by macrophages was observed using a fluorescence microscope (scale bar: 200 μm). (E) RT‐qPCR showed elevated miR‐196a‐5p levels in exosomes and macrophages after electroporation. (F) RT‐qPCR was performed to detect the mRNA expression levels of macrophage polarization markers (iNOS and CD86 for M1 markers; CD206 and CD163 for M2 markers) in EMs exosomes and EMs exosomes@miR‐196a‐5p. (G) Western blot was used to detect the protein expression levels of macrophage polarization markers (iNOS and CD86 for M1 markers; CD206 and CD163 for M2 markers) in EMs exosomes and EMs exosomes@miR‐196a‐5p. (H) The proportion of CD86+ (M1) and CD206+ (M2) macrophages in EMs exosomes and EMs exosomes@miR‐196a‐5p was detected using a flow cytometer. Data represent mean ± SD from three independent experiments. EMs exosomes@miR‐196a‐5p: EMs patient‐derived exosome‐encapsulated miR‐196a‐5p mimic. *: *P* < 0.05, **: *P* < 0.01, ***P < 0.001, compared with EMs exosomes.

The miR‐196a‐5p mimic was electroporated into EMs exosomes and then applied to macrophages. First, TEM, NTA, and Western blot confirmed that there was no significant difference between electroporated EMs exosomes and nonelectroporated EMs exosomes (Figure 4A–C). They could also be taken up by macrophages (Figure 4D). RT‐qPCR results showed that the miR‐196a‐5p expression level was elevated in exosomes after electroporation, whereas after EMs patient‐derived exosome encapsulated miR‐196a‐5p mimic (EMs exosomes@miR‐196a‐5p) acted on macrophages, miR‐196a‐5p expression in macrophages in the EMs exosomes@miR‐196a‐5p group, compared to the EMs exosomes group, was elevated (Figure 4E). Meanwhile, the expression of M1 macrophage markers in the EMs exosomes@miR‐196a‐5p group increased, whereas the expression of M2 macrophage markers decreased (Figure 4F,G). FCM results showed that compared to the EMs exosomes group, the proportion of M1 macrophages in the EMs exosomes@miR‐196a‐5p group increased, whereas the proportion of M2 macrophages decreased (Figure 4H). These results indicate that miR‐196a‐5p carried by exosomes enters macrophages and affects their polarization.

### 3.5 EMs exosomes encapsulate miR‐196a‐5p to activate the Hippo pathway in macrophages

**FIGURE 5:**
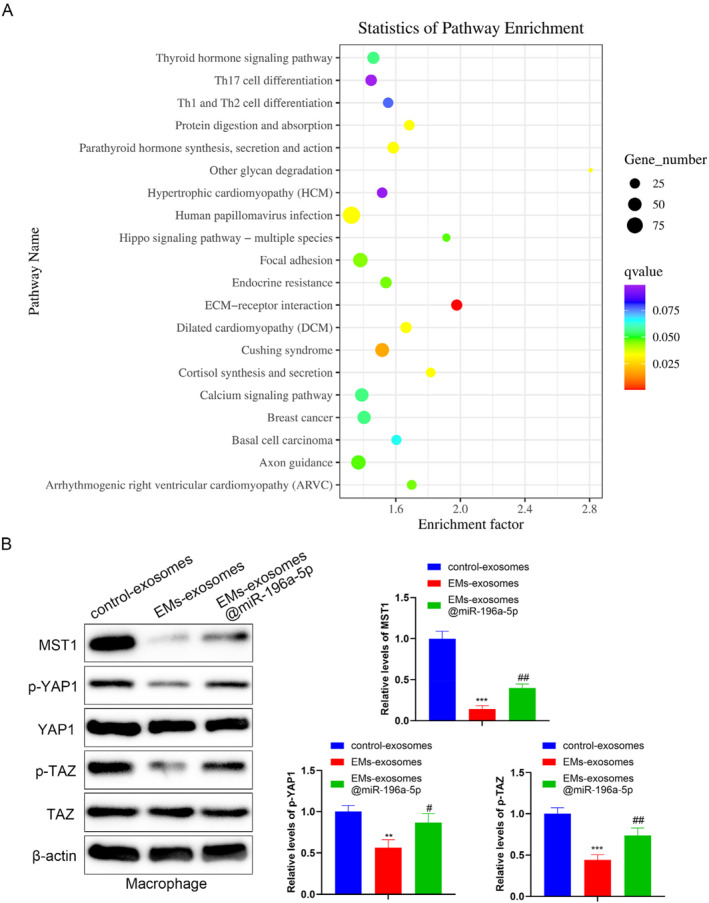
Effect of encapsulated miR‐196a‐5p in exosomes on the Hippo pathway in macrophages. (A) Kyoto Encyclopedia of Genes and Genomes (KEGG) pathway analysis was carried out on the predicted target genes using bioinformatics tools. (B) Western blot was used to detect Hippo pathway‐related proteins (MST1, p‐YAP1, YAP1, p‐TAZ, and TAZ). Data represent mean ± SD from three independent experiments. *: *P* < 0.01, ***: *P* < 0.001, compared with control exosomes; ^#^: *P* < 0.05, ^##^: *P* < 0.01, compared with EMs exosomes.

The results of the KEGG pathway enrichment analysis for differentially expressed miRNA target genes are shown in Figure 5A, which presents the top 20 pathways with the most significant differences. Among them, we identified the Hippo pathway, which has been reported in previous studies to be involved in the polarization of macrophages.^19^, ^20^ To verify whether the Hippo pathway undergoes changes, we treated macrophages with exosomes from different groups. The Western blot results showed that compared with the control exosomes group, the expression levels of MST1, p‐YAP1, and p‐TAZ were decreased in the EMs exosomes group, whereas EMs exosomes@miR‐196a‐5p promoted the expression levels of MST1, p‐YAP1, and p‐TAZ (Figure 5B). These results suggest that EMs exosomes encapsulating miR‐196a‐5p may activate the Hippo pathway in macrophages.

### 3.6 Inhibiting the Hippo pathway promotes M2 macrophage polarization

**FIGURE 6:**
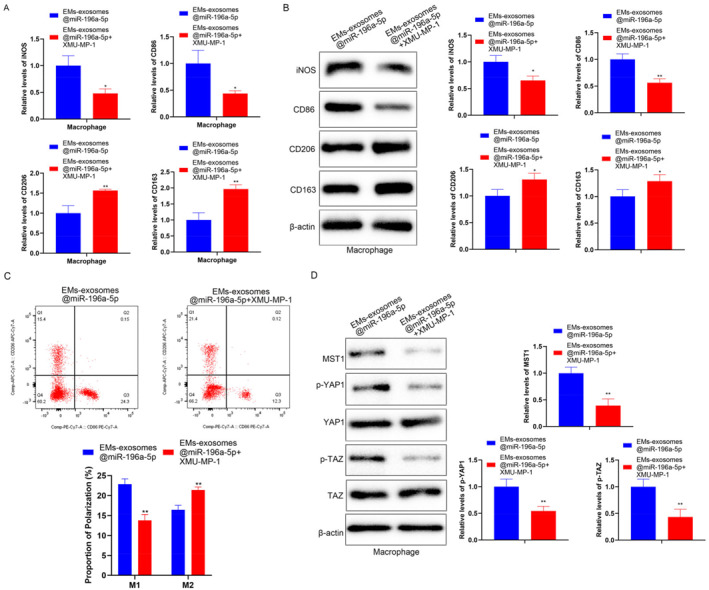
The effect of the Hippo pathway on macrophage polarization. To investigate whether EMs exosomes@miR‐196a‐5p affects macrophage polarization through the Hippo pathway, we treated macrophages with EMs exosomes@miR‐196a‐5p in combination with the Hippo pathway inhibitor XMU‐MP‐1. (A) RT‐qPCR was used to detect macrophage polarization markers (iNOS and CD86 for M1 markers; CD206 and CD163 for M2 markers). (B) Western blot was used to detect the protein expression levels of macrophage polarization markers (iNOS and CD86 for M1 markers; CD206 and CD163 for M2 markers). (C) The proportion of CD86+ (M1) and CD206+ (M2) macrophages was detected using a flow cytometer. (D) Western blot was used to detect Hippo pathway‐related proteins (MST1, p‐YAP1, YAP1, p‐TAZ, and TAZ). Data represent mean ± SD from three independent experiments. XMU‐MP‐1: Hippo inhibitor. *: *P* < 0.05, **: *P* < 0.01, compared with EMs exosomes@miR‐196a‐5p.

To investigate whether EMs exosomes@miR‐196a‐5p affects macrophage polarization through the Hippo pathway, we treated macrophages with EMs exosomes@miR‐196a‐5p in combination with the Hippo pathway inhibitor, XMU‐MP‐1. RT‐qPCR and Western blot results showed that compared to the EMs exosomes@miR‐196a‐5p group, the expression of M1 macrophage markers (iNOS and CD86) decreased, whereas the expression of M2 macrophage markers increased in the EMs exosomes@miR‐196a‐5p + XMU‐MP‐1 group (Figure 6A,B). FCM results indicated an elevated proportion of M2 macrophages in the EMs exosomes@miR‐196a‐5p + XMU‐MP‐1 group (Figure 6C). Western blot results demonstrated a decrease in the expression of Hippo pathway‐related proteins in macrophages from the EMs exosomes@miR‐196a‐5p + XMU‐MP‐1 group (Figure 6D). These results confirm that EMs exosomes encapsulating miR‐196a‐5p inhibit M2 macrophage polarization by activating the Hippo pathway.

### 3.7 M2 type macrophages promote viability and metastasis of endometriosis cells

**FIGURE 7:**
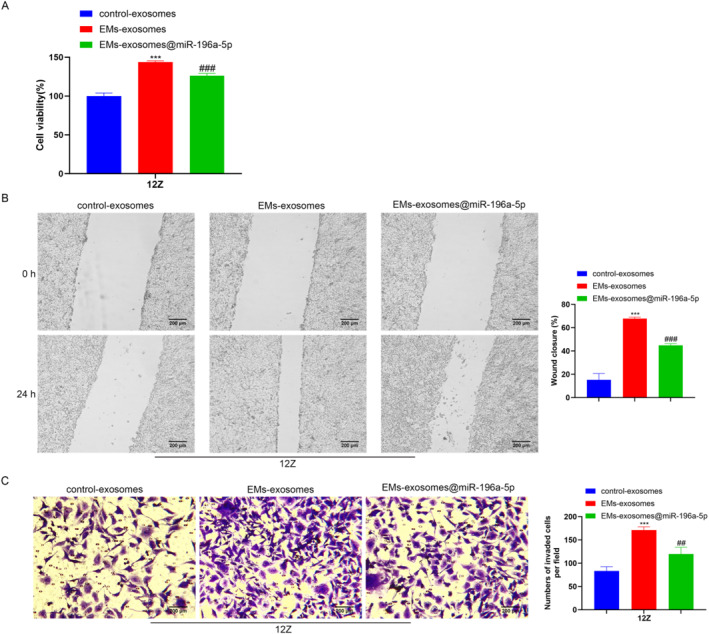
Role of macrophage polarization on 12Z cells. To investigate the impact of macrophages on endometriosis cells, we co‐cultured macrophages from different groups with 12Z cells using cell chambers. (A) CCK‐8 was used to assess the growth viability of 12Z cells. (B) Scratch assay was performed to detect the migration capacity of 12Z cells (scale bar: 200 μm). (C) A Transwell invasion assay was used to analyze the invasion capacity of 12Z cells (scale bar: 200 μm). Data represent mean ± SD from three independent experiments. ***: *P* < 0.001, compared with control exosomes; ^##^: *P* < 0.01, ^###^: *P* < 0.001, compared with EMs exosomes.

To investigate the impact of macrophages on endometriosis cells, we co‐cultured macrophages from different groups with 12Z cells using cell chambers. CCK‐8 results suggest that, compared to the control exosomes group, the EMs exosomes‐treated macrophages promote the growth viability of 12Z cells, whereas the EMs exosomes@miR‐196a‐5p group macrophages inhibit the proliferation ability of 12Z cells (Figure 7A). Cell metastasis ability was analyzed using scratch and invasion assays, and the results showed that, compared to the control exosomes group, the EMs exosomes‐treated macrophages promote the migration ability of 12Z cells, whereas the EMs exosomes@miR‐196a‐5p group macrophages correspondingly inhibit the migration ability of 12Z cells (Figure 7B,C). These findings indicate that M2 macrophages exacerbate the condition of endometriosis.

## 4 DISCUSSION

Endometriosis profoundly impacts women's health and quality of life.^21^ However, because of its complex course and unclear pathogenesis, it continues to perplex clinical medical practitioners.^22^ Therefore, discovering its pathogenesis and finding its diagnostic and therapeutic targets and methods are particularly important.

Macrophages are an important subset within the immune cell population, playing a significant role in mediating inflammatory responses and phagocytosis.^23^ Macrophages mainly exist in two activation states, M1 and M2. M1 macrophages primarily secrete proinflammatory factors and actively participate in immune responses, serving an immunosurveillance role, whereas M2 macrophages mainly secrete anti‐inflammatory factors and primarily exert immunosuppressive functions.^24^ In previous studies, the functional imbalance of macrophages was an important factor leading to the occurrence and development of endometriosis, M2 macrophages had been reported to mainly participate in and promote the progression of endometriosis.^25^, ^26^ Therefore, how to regulate macrophage polarization toward a beneficial direction can be considered an important measure for treating and improving endometriosis. In our study, we found that exosomes derived from EMs could promote M2 macrophage polarization, and M2‐polarized macrophages, in turn, facilitate the proliferation and metastasis of endometriosis cells. This further underscores the proendometriotic role of M2 macrophages.

Exosomes, as extracellular vesicles secreted by cells, contain a variety of biological components, such as miRNA, peptides, etc.^27^ This further underscores the proendometriotic role of M2 macrophages.^28^ A study had reported that miR‐500a‐5p, a microRNA secreted by tumor‐associated fibroblasts in exosomes, could promote the proliferation and metastasis of breast cancer cells.^29^ In endometriosis, a clinical study reported that the miRNA expression profile in exosomes derived from patients with endometriosis shows a certain correlation with the severity of the patient's disease.^30^ In our study, we found that miRNAs with significantly differential expression also exist in exosomes extracted from the serum of both healthy control and patient groups through miRNA sequencing.

In recent years, exosomes have gradually developed into a good delivery system due to their high cost‐effectiveness and good safety, mainly delivering substances including drugs, siRNA, miRNA, proteins, etc.^31^, ^32^ We encapsulated miR‐196a‐5p into exosomes through electroporation and found that it could be phagocytosed by macrophages, enhancing the expression level of miR‐196a‐5p in macrophages and reversing the M2 polarization effect of exosomes derived from the serum of patients with endometriosis on macrophages. Furthermore, macrophages treated with the EMs exosome@miR‐196a‐5p group alleviated the proliferation and metastasis of 12Z cells. This indicated that exosomes can serve as a good drug encapsulation and delivery system for the subsequent treatment of diseases, including endometriosis. A research report published in 2024 stated that miR‐146a‐5p contained in exosomes secreted by ectopic endometrial stromal cells promotes M2 macrophage polarization through TRAF6, exacerbating the symptoms of endometriosis.^33^ Although the study reported results similar to ours, it did not explore the specific molecular mechanisms of macrophage polarization. In our research, through KEGG analysis combined with Western blot detection, we found that the occurrence of macrophage polarization may be related to the Hippo pathway. Upon integrating the Hippo pathway inhibitor with the synergistic effect of EMs exosome@miR‐196a‐5p, it was revealed that EMs exosome@miR‐196a‐5p has the capability to stimulate the Hippo pathway, thereby facilitating the polarization toward M1‐type macrophages. Conversely, the Hippo pathway inhibitor, by dampening the pathway, concurrently hindered the polarization of M1‐type macrophages and fostered the shift toward M2‐type macrophages. This indicated that the Hippo pathway may serve as a potential key pathway for modulating macrophage dysfunction, thereby playing a role in improving the symptoms of endometriosis. In other studies, the Hippo pathway had also been confirmed to be involved in macrophage polarization.^34^, ^35^, ^36^

miR‐196a‐5p, as a classic noncoding RNA, plays a role in numerous biological processes. For example, miR‐196a‐5p played an important role in the progression from chronic atrophic gastritis to gastric cancer.^37^ Similarly, high expression of miR‐196a‐5p was highly correlated with the malignant biological behavior of glioma.^38^ It was noteworthy that researchers had found that miR‐196a‐5p promotes the progression of endometrial cancer by regulating FOXO1.^39^ Our research findings indicated that the expression of miR‐196a‐5p is decreased in exosomes derived from the serum of patients with endometriosis. However, delivering miR‐196a‐5p via exosomes to macrophages could promote M1 polarization of macrophages and inhibit the proliferation and metastasis of 12Z cells. This suggests that miR‐196a‐5p is a potential therapeutic target for the diagnosis and treatment of endometriosis.

## 5 CONCLUSION

Our findings suggest that exosomal miR‐196a‐5p alleviates endometriosis by promoting M1 macrophage polarization via Hippo pathway activation. Although this study confirmed the relationship between exosomes, miR‐196a‐5p, macrophage polarization, and endometriosis, there are certain limitations due to the lack of in vivo mouse model experiments. Although sequencing and RT‐qPCR confirmed reduced exosomal miR‐196a‐5p levels and subsequent overexpression studies, the lack of miR‐196a‐5p inhibition experiments is a study limitation. Additionally, which mRNA miR‐196a‐5p functions through is a topic that needs further detailed investigation. Exploring the specific downstream signaling molecules and regulatory networks of the Hippo pathway also deserves subsequent in‐depth studies. Despite these limitations of this study, our findings provide a possible theoretical basis for the clinical diagnosis and treatment of endometriosis.

## AUTHOR CONTRIBUTIONS

**Bin Lu:** Conceptualization; writing—original draft; funding acquisition; data curation. **Qixiang Huang:** Supervision; investigation; resources; visualization. **Yanyu Zhong:** Writing—review and editing; conceptualization, project administration. All authors were involved in drafting and revising the manuscript.

## CONFLICT OF INTEREST STATEMENT

The authors declare no conflicts of interest.

## ETHICS STATEMENT

This study conformed to the principles outlined in the Declaration of Helsinki and was approved by the Ethics Committee of Wuhu No. 1 People's Hospital (No. YYLL20220089). Written informed consent was obtained from all the participants before enrollment.

## Data Availability

All data generated or analyzed during this study are included in this published article.
